# Oncological and functional results after surgical treatment of bone metastases at the proximal femur

**DOI:** 10.1186/s12893-018-0336-0

**Published:** 2018-01-25

**Authors:** Grzegorz Guzik

**Affiliations:** 1 Department of Orthopaedic Oncology, Specialist Hospital in Brzozów- Podkarpacie Oncology Centre, Bielawskiego 18, 36-200 Brzozów, Poland; 2 Korczyna, Poland

**Keywords:** Bone metastasis, Proximal femur, Modular endoprostheses, Tumors resections

## Abstract

**Background:**

Metastatic lesions to the proximal femur occur frequently (about 10% of patients with cancer) and require surgical treatment. There are many surgical methods of treatment, however, use of the tumor modular endoprostheses seems to be particularly promising. The aim of study was to evaluate oncological and functional results of treatment in patients with proximal femur metastases. Oncological results were evaluated considering the survival of patients and the number of local recurrences. Functional results were assessed as pain intensity in VAS score and performance in Karnofsky and MSTS score.

**Methods:**

Between 2010 and 2016, 122 patients with metastatic tumour to the proximal femur were treated in our hospital. Majority of the patients were women − 77 patients. The mean age was 67 years for women and 72 years for men. Pathological fracture was diagnosed in 98 cases. Metastatic bone tumors commonly develop from breast cancer – 48 and myeloma – 24. One hundred one patients underwent tumor resection and in 21 cases metastatic tumors was not resected. In 75 patients wide tumour resection and modular endoprosthetic replacement were prefomed. Twenty-one patients underwent standard or long stem hip endoprosthetic replacement. Intramedullary gamma nails were implanted in 20 cases and DHS plate in 6 cases. In 92 cases 3-4 weeks after surgery patients undergo external beam radiotherapy (8Gy).

Functional results were assessed as pain intensity in VAS score and performance in Karnofsky and MSTS score. Oncological results were evaluated considering the survival of patients and the number of local recurrences.

**Results:**

The mean follow-up of patients was 27 months (min 4, max 51). Forty-five patients died before last visit in hospital. The mean survival after modular endoprosthetic replacement was 860 days and after bone fixations 360 days. We noticed 9 cases of local recurrences or progressions, 6 in patients who had no radiotherapy. Three patients after modular endoprosthesis replacement and 6 after bone fixations.

After surgery, all patients experienced improvement in the comfort of life resulting from reduction in pain. Mean VAS score before modular endoprosthetic replacement was 6.8 and after 3.4; before standard prothesoplasty 4.9 and after 2.8; and before and after bone fixation 6.9 -5.1. Mean MSTS score was respectively 6.4-19.8; 8.8-22.4 and 10.8-18.2.

In 6 patients after modular endoprosthesis replacement, delayed wound healing were observed. Infectious complications were not observed after fixation with nails and plates. In 3 cases, the fixation was failed. The systemic complications affected 12 patients.

**Conclusions:**

Results of surgical treatment for metastases to the proximal femur are particularly good in patients after standard or modular endoprostheses replacement. The author considers this treatment method to be optimal in patients with good prognosis.

## Background

Metastatic lesions located in the proximal femur are particularly frequent. About 10% of patients with primary malignant tumors will develop metastasis of proximal femur. Among femur metastatic tumors, 50% of the lesions occur in the femoral neck, 30% occur in the subtrochanteric site, and 20% occur in the intertrochanteric site. This is related to the well-developed vascular system in the intertrochanteric area [1–5]. Most frequently bone metastases are derived from breast, kidney, thyroid, prostate cancer, or myeloma. Apart from prostate cancer, most of metastases are lytic or mixed, and thus patients are at a high risk of pathological fractures [1–5]. Radiographs show bone metastasis, and allow evaluation of a risk of pathological fracture, according to the Mirels scale. Mirels proposed a scoring system based on four characteristics: site of lesion; nature of lesion; size of lesion; and pain. A score from 1 to 3 is assigned to each variables. Overall score greater 8 point indicated prophylactic internal fixation prior to irradiation. Sometimes, computer tomography or magnetic resonance scans are performed before the surgery, to precisely evaluate the extent of the lesion, and infiltrations to soft tissues, particularly vessels and nerves [1, 2].

CT scans or PET is also applied to the staging of diseases in tumor patients. The TNM system is the most widely used cancer staging system. The stage of the cancer allow to develop a prognosis and design a treatment plan for individual patients. In most cases the stage is based on four main factors: location of the primary tumor, size of tumor, lymph node involvement, presence or absence of distant metastasis. Qualification for treatment considers patient’s age and general condition, as well as cancer type, malignancy and staging. Capanna and Campanacci introduced in 2001 an algorithm of metastasis of long bone and pelvis treatment. The patients was divided into 4 classes: 1- solitary lesion with good prognosis; 2- pathologic fracture; 3- impending fracture and 4 other lesions [5–7]. In selecting the adequate treatment in long bones and pelvis, important parameters are: expected survival, the type, visceral metastasis, the time interval from the primary lesion, the risk of pathological fracture, and the sensitivity to chemotherapy, hormone therapy, and irradiation. Qualification for treatment should be multidisciplinary. The oncological team consists of an oncologist, radiotherapist and orthopaedic surgeon. To determine patients’ life expectancy and prognosis Karnofsky, ECOG and SSG scores can be used [1, 6–10].

Patients in a generally good condition and with good prognosis undergo resection of the metastatic tumour. Radical resection of the metastases significantly reduces a number of local recurrences. Many studies also indicate overal survival and quality of life improvement after radical metastases resections [9–11]. The removed fragment of the bone is supplemented using various methods. They include intramedullary nailing and plates combined with bone cement (PMMA). Also use of tumour modular endoprostheses for treatment of metastases increases. When metastases are small, standard prostheses can be implanted; however, majority of patients require special prostheses: long-shaft or modular endoprostheses [3, 6, 11, 12].

In patients with severe condition, advanced cancer, and suffering with comorbidities, and with poor life expectancy, metastatic tumours are not resected and only palliative fracture fixation is performed. In these cases, metastatic tumor progression can be expected [7, 8, 13].

Surgeries performed in cancer patients are procedures at a high risk of thromboembolic and infectious complications. Radiotherapy is not an alternative to surgical treatment, however in many cases can reduce pain intensity. Post-surgery radiotherapy is a particularly valuable method reducing a risk of local recurrence [1–4, 14, 15].

The aim of study was to evaluate oncological and functional results of treatment in patients with proximal femur metastases. Oncological results were evaluated considering the survival of patients and the number of local recurrences. Functional results were assessed as pain intensity in VAS score and performance in Karnofsky and MSTS score.

## Methods

Between 2010 and 2016, 122 patients with metastatic tumour to the proximal femur were treated in our hospital. Basic patients’ medical records were analysed, with particular focus on the cancer type, disease duration, treatment type, cancer staging and prognosis. The analysis include pre- and post-operative radiograms, surgery course and type, complications related to the surgery and their causes, and applied treatment. Qualification for treatment always was multidisciplinary with the participation of oncological team. To determine patients’ life expectancy and prognosis Karnofsky, ECOG and SSG scores were used.

Majority of the patients were women (77 patients), with 45 men. The mean age was 67 years for women and 72 years for men. A pathological fracture was diagnosed in 98 cases, in 24 cases, the size of the metastasis implied a high risk of a fracture. A large soft tissues tumor was found in 65 patients. Single metastases were diagnosed in 36 patients mainly with breast cancer - 18, kidney cancer - 8, myeloma − 7 and thyroid cancer - 3 patients. In 86 cases multiple bone metastases were diagnosed and located mainly in the spine and pelvis. The number of metastases, as a single factor, did not decide on the method of surgical treatment. Metastatic bone tumors commonly develop from breast cancer - 48, myeloma - 24, kidney cancer - 19, bowel cancer - 3, thyroid cancer - 4, lung cancer - 5, prostate cancer − 3, and unknown primary - 16 cases.

One hundred one patients underwent tumor resection and in 21 cases metastatic tumors was not resected. The indications for radical metastasis resection and modular endoprosthesis implantation were good patients general condition and prognosis regarding life expectancy. Tumors were not resected in patients with a severe general condition and survival prognosis shorter than 3 months.

In 75 patients wide tumour resection and modular endoprosthetic replacement were prefomed. Cemented proximal femur modular stem was used in 22 cases (GMRS-Stryker) and cementless in 53 cases (MUTARS-Implant Cast). In patients with poor bone quality or after resections in “femoral isthmus” cemented stems were used, because stable cement less stems implantation in the place where the bone expands is impossible. In 68 cases bipolar cup and in 7 cases cemented cup was used. Bipolar cups were used if no degenerative joint disease were observed. In cases with hip osteoarthritis or with metastases in the acetabulum cemented cups were used. In 8 cases Treviera mesh for reattachment of soft tissue, especially muscle was used. Mean range of bone resection was 160 mm. (from 100 to 200 mm).

Fourteen patients underwent intralesional tumour resection and standard hip endoprosthetic replacement, and 7 patients long stem endoprosthetic replacement (Stryker). When metastatic tumour were localised in the femoral head or neck standard endoprostheses were used. If the tumour spread on intertrochanteric region without cortical bone damage long stem were used. In our series only cemented standard endoprostheses were used.

Intramedullary gamma nails (Synthes) were implanted in 18 cases without metastasis resection and in 2 cases after intralesional tumour resection with the defect of bone filled with bone cement (PMMA). In 6 cases the metastases was intralesional resected and the bone was fixed with the DHS plate (Synthes) with PMMA. Bone fixations was performed in cases with poor general condition and life expectancy less than 3 months. Type of bone fixation depend on fracture location and shape, extent of bone defect and surgeon preferences. Type of cancers and methods of treatment was summarized in Table 1.

**Table 1:** Cancer characteristics and applied treatment *N* = 122

Cancer	N (%)
Breast cancer	48 (40)
Myeloma	24 (20)
Kidney cancer	19 (16)
Colon cancer	3(2)
Thyroid cancer	4 (3)
Lung cancer	5 (4)
Prostate cancer	3 (2)
Unknown	16 (13)
Treatment	
Megaprosthesis	75 (61)
Standard Prosthesis	21 (17)
Gamma nail	20 (17)
DHS	6 (5)

Results are presented as a number with a percentage

Ninety-two patients undergo external beam radiotherapy in 3-4 weeks after surgery according to the EMSOS recommendation. The decision about necessity of radiotherapy application was made by the oncological consilium. It was not used when radiation dose was exceeded prior to surgery or in cases with contraindications for radiotherapy. In our series 8 Gy in single dose was usually used. 30-40 Gy in 10 fractions was not used becouse of the risk of skin necrosis and infectious complications. Higher doses of radiation were used in patients treated only with radiotherapy. Radiotherapy was performed in 75 patients after modular endoprosthetic replacement, 4 patients after standard endoprosthetic replacement and 13 patients after bone fixation.

Functional results were assessed as pain intensity in VAS score and performance in Karnofsky and MSTS score. The analysis focused on walking status, necessary orthopaedic equipment, muscle capacity and joint mobility were performed. Oncological results were evaluated considering the survival of patients and the number of local recurrences and metastatic tumor progressions.

Quantitative variables were expressed as means (x) with standard deviations. To compare effects of different treatments options paired Student’s t-test was used. The inter-group differences were tested using one-way ANOVA. The categorical variables were expressed as percentages. The inter-group differences were tested using the χ2 test. All statistical analyses were performed by using Statistica 10. A value of *P* < 0.05 was considered statistically significant. Survival data was estimated with Kaplan-Meier curve. Survival in patient groups was estimated with Log-rank test.

The research has been performed in accordance with the declaration of Helsinki. As this retrospective analysis consists of anonymised clinical routine data, the Research Ethics Committee deems the application for and issue of an Ethics approval not necessary. All the patients gave a written consent to the use of data for research. Name of Ethics committee: Ethics Committee in Cracov, ul Krupnicza 11a 31-123 Cracov, tel. + 48126191712, fax + 48124225755.

## Results

**Fig. 1:**
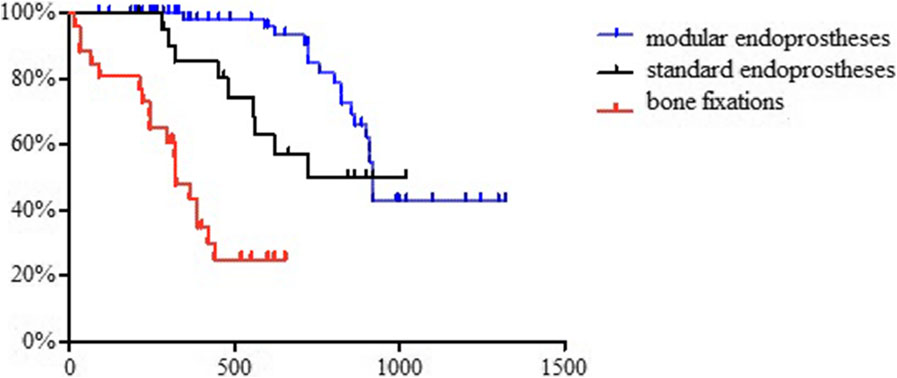
Survival In patients after different methods of surgery

The mean follow-up of patients was 27 months (min 4, max 51). Forty-five patients died before last visit in hospital. Survival data was estimated with Kaplan-Meier curve – Fig. 1. The mean survival after modular endoprosthetic replacement was 860 days and after bone fixations 360 days. The causes of death were progression of cancer disease – 42 patients. In 4 patients it was circulation problems and in 3 pulmonary embolism. In 8 cases direct cause of death was not recognized.

Nine cases of local recurrences or metastatic tumour progressions were observed. Six cases concerned patients who had no radiotherapy and 3 despite radiotherapy. Local recurrance was observed in 3 patients after modular endoprosthetic replacement and metastatic tumour progression in 6 patients after bone fixations. In one patient prosthesis was loosened after 4 months. In other cases after palliative radiotherapy the radiological and clinical status seems to be stable.

Before surgery patients’ performance were evaluated according to the Karnofsky, MSTS scale, and the pain intensity was assessed according to the VAS scale – Tables 2 and 3. Patients with femur fractures were not able to walk. The limb was placed in a forced position, with various deformations (shortening, bent axis, thickened contour). Any attempts at movement resulted in pain. No signs of ischaemia or damage to peripheral nerves were observed. Patients with extensive lytics lesions in the lower limb walked supported on a walking frame. The joint function was limited by pain. Lifting the leg above the bed with a knee straightened when lying caused pain or was impossible.

**Table 2:** Mean results of MSTS and Karnofsky performance score in patients before and after the surgery, in different treatment method

Treatment options	MSTS score		Karnofsky score	
	Before intervention	After intervention	Before intervention	After intervention
A) Megaprosthesis	6.4 ± 0.4	19.8 ± 0.6*	53 ± 7	67 ± 9*
B) Standard prosthesis	8.8 ± 1.2	22.4 ± 0.6*	55 ± 9	70 ± 4*
C) Gamma Nail and DHS	10.8 ± 0.8	18.2 ± 1.0*	50 ± 10	55 ± 7
Significant differences intergroup	NS	Gr. C > A/B*	NS	Gr. C > A/B*

Results are presented as a mean ± standard deviation

*p < 0.05

**Table 3:** Mean results of VAS score in patients before and after the surgery, in different treatment method

Treatment options	VAS score		VAS score
	Before intervention		After intervention
A) Megaprosthesis	6.8 ± 0.5		3.4 ± 0.8*
B) Standard prosthesis	4.9 ± 1.1		2.8 ± 0.6*
C) Gamma Nail and DHS	6.9 ± 0.7		5.1 ± 1.0*
Significant differences intergroup	NS	Gr. C > A/B*	

Results are presented as a mean ± standard deviation

*p < 0.05

**Fig. 2:**
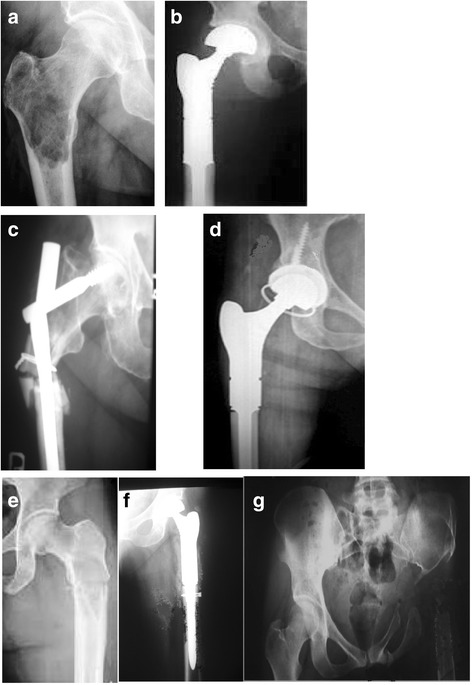
Preoperative and postoperative radiograms series. **a** Proximal femur breast cancer metastasis, **b** radiogram after tumour resection and modular prosthesis implantation. **c** Proximal femur prostate cancer metastasis and fracture after stabilisation with use of gamma nail. Fail of fixation - gamma nail was broken, **d** after proximal femur resection and modular prosthesis implantation. **e** Proximal femur renal cancer metastasis, **f** local recurrence after tumor resection and endoprosthesis implantation, **g** radiogram after prosthesis removal

After surgery, all patients experienced improvement in the comfort of life resulting from reduction or resolving of pain. Fourteen days after surgery, pain intensity and patients’ performance were evaluated - Fig. 2.

After 3 months from surgery, walking recovery were evaluated together with a need for using crutches or a walking frame. Forty-one patients walks effectively without crutches, 44 patients uses 1 crutch or a stick when walking for longer distances, while 27 patients uses 2 crutches, and 10 patients walks with a walking frame – Table 4.

**Table 4:** Functional status after 3 months follow up depending on treatment option

Functional status	Treatment options		
	Megaprosthesis	Standard prosthesis	Gamma Nail / DHS
	*n* = 75	*n* = 21	*n* = 26
Walking without crutches	23	18	0*
Walking with 1 crutch	41	3	0*
Walking with 2 crutches	7	0	20*
Use of a walking frame	4	0	6*

Results are presented as a number with a percentage

*p < 0.05 χ^2^

When modular prostheses were implanted, a positive Trendelenburg’s sign was observed in all cases, implying impaired function of gluteal muscles. Patients could walk on stairs using step-over-step (46 patients) or step-by-step (29 patients) patterns. The patients experienced particular problems with thigh abduction when standing (48 patients) and with lifting the limb to the side when lying on a healthy side (47 patients).

In 2 patients after standard or long-stem endoprosthesis replacement, gluteal muscle failure was observed.

After internal fixation with intramedullary nails and plates, no signs of gluteal muscle failure were observed, but the hip joint mobility was significantly limited. In majority of patients, axial pressure on the operated limb increased pain intensity.

No complications were observed after standard endoprosthesis replacement. No prosthesis dislocation was observed. In 6 patients after modular endoprosthesis replacement, delayed wound healing were observed, with discharge from the wound. In 4 cases, revision procedures were performed. The wound was cleaned of granulation tissue, rinsed, and a garamycin sponge was implanted. The endoprosthesis was not removed. Intraoperative cultures were negative. The patients recovered without any further problems. In one case it was necessary to remove the endoprosthesis, and after debridement a one stage realloplasty was performed. After the revision surgery, the wound healed within 2 weeks.

Infectious complications were not observed after fixation with nails and plates. In 3 cases, the fixation was failed - 5, 7, and 8 months after the surgery.

In one patient the intramedullary nail broke. Patients underwent bone resection within the healthy tissues and modular endoprosthesis replacement. The systemic complications affected 12 patients. Six patients suffered from pulmonary embolism, and their condition improved quickly. Three patients suffered from myocardial infarction in the postoperative period. Three patients were temporary dialysed due to kidney failure.

## Discussion

The majority of metastases of the long bones affect the proximal femur. Other locations are not as frequent, and metastases located below the elbow or knee joint are rare. The location in epiphyses and metaphyses results from a very well developed vascular system in these parts of the bone. Oncological patients rarely are provided care by an orthopaedic specialist, and oncologists do not recognize signs indicating development of a metastasis, as they are often discrete. Usually, diagnosis is made only when symptoms of pathological bone fracture develop [1, 2, 4].

The radiotherapy had limited importance as a single method of treatment. Radiotherapy is useful for treatment metastases to flat bone and patients with contraindication for surgical treatment. As adjuvant therapy reduce risk of local recurrence, progression and pain. However surgical treatment after radiation have increased risk of delayed wound healing and infection. There are many methods of bone metastases radiotherapy. In patients who was previously radiated and in cases of uncoplicated bone metastases 8Gy in single dose should be usually used. In cases with pathological farcture and treated only with radiotherapy 30-40 Gy in 10 fractions can be used [15–18].

Surgical treatment for pathological fractures of the long bones is a therapy of choice. Usually, intramedullary nails and titanium plates are used. A site of removed metastasis is filled with bone cement (PMMA). Outcome of this treatment varies. Piccioli et al. in their study reported good functional outcomes in group of 80 patients with the proximal femur pathologic fractures treated with a titanium proximal nail. All patients reported pain relief and improvement in the quality of live. The patients’ survival rate were 40% at 1 year, 25% at 2 years and 15% at 3 years. Authors concluded that intramedullary nailing should be reserved for pathologic fractures when cancer is in an advanced stage. In patients with good prognosis intramedullary nail fixation may fail or implant breaking occurs. In such cases repeated surgery is required, increasing a risk of complications, particularly infectious ones. Even successful fixations rarely enable patients to bear weight on the operated limb [13, 19–21].

Use of modular prostheses for treatment of metastatic lesions becomes increasingly justified due to potentially long expected survival of a patient with metastases of breast, prostate, kidney, bowel, or thyroid cancer, or myeloma. The optimum results for treatment of metastatic lesions are achieved in patients without actual pathological fracture, where tumour resection was performed with a wide margin of healthy tissues. In these patients local recurrences, implant damages or hazardous perioperative complications are less frequently observed. The mean survival of patients after radical removal of a bone metastasis may even reach 37 months, and, varies in different cancers, depending on cancer malignancy, a disease stage, and a treatment method. The recurrence rate after resection of metastatic tumours located within the long bones ranges from 4 to 28% [9–11, 22–26].

Also, it is very important if cemented or cement less endoprostheses are used. Pala et al. reported 60 months overal survival for patients treated with use of cemented endoprostheses - 64% and cementless endoprostheses - 78%. Survival to infection was 68% and 82% and survival to aseptic loosening 94% and 96% respectively [27–30].

Oncological patients represent the group at the highest risk of infections and thromboembolic complications. A rate of described infectious complications ranges from 1.2%–19.5%. One of the most important factors related to the infection risk is preoperative radiotherapy. After radiation, tissues are particularly fragile, necrotic, with damaged blood vessels. It also results in chronic ischaemia of skin and muscles. Reduced immunity, anaemia and coagulation disorders are also observed directly after chemotherapy [3, 8].

Other frequently described complications include prosthesis dislocations and loosening. A high risk of dislocations results from damage to muscle attachments and lack of dynamic joint stabilisation. Even when muscles are sutured back to special attachment points on prostheses or meshes, their full capacity and power is not restored. Additionally, some muscles are usually removed during tumour resection, and the new attachment is rarely in its typical anatomical location. The estimated rate of revision procedures is 3–17% [27–29].

In our material oncological and functional outcome after standard and modular endoprosthetic replacement were good. Impairment in the gluteal muscles function is not particularly problematic. The majority of patients walk without crutches. Slight difference in the lower limbs length is not noticed by patients, and does not affect their walking. Complications after endoprotesoplasty were rarely observed.

## Conclusions

Patients with metastases to the proximal femur if general health condition allows should be used treated surgical. Radical resection of the metastatic tumours gives good treatment results. This protects against local recurrence, and loosening or damage to the implant. Treatment with use of prostheses shows the best outcome. Pain intensity and patients performance improved significant. Majority of patients recovery walking ability.

## Abbreviations

DHS: Dynamic Hip Screw
ECOG: Eastern Cooperative Oncology Group
EMSOS: European Musculo-Skeletal Oncology Society
PMMA: Polymethyl methacrylate
SSG: Survival Scoring System Scandinavian Sarcoma Group
VAS: Visual Analogue Scale
